# In Vitro Nephrotoxicity and Permeation of Vancomycin Hydrochloride Loaded Liposomes

**DOI:** 10.3390/pharmaceutics14061153

**Published:** 2022-05-28

**Authors:** Nicole Papp, Jeffin Panicker, John Rubino, Gwendolyn Pais, Alexander Czechowicz, Walter C. Prozialeck, Brooke Griffin, Volkmar Weissig, Marc Scheetz, Medha D. Joshi

**Affiliations:** 1 Department of Pharmaceutical Sciences, Midwestern University, 555 31st Street, Downers Grove, IL 60515, USA; npapp95@midwestern.edu (N.P.); jeffin.panicker@midwestern.edu (J.P.); jrubino39@midwestern.edu (J.R.); 2 Department of Pharmacy Practice, Midwestern University, 555 31st Street, Downers Grove, IL 60515, USA; gpais@midwestern.edu (G.P.); bgriff@midwestern.edu (B.G.); mschee@midwestern.edu (M.S.); 3 Department of Pharmaceutical Sciences, Midwestern University, 19555 N. 59th Avenue, Glendale, AZ 85308, USA; alexander.czechowicz@midwestern.edu (A.C.); vweiss@midwestern.edu (V.W.); 4 Department of Pharmacology, Midwestern University, 555 31st Street, Downers Grove, IL 60515, USA; wprozi@midwestern.edu

**Keywords:** vancomycin hydrochloride, in vitro toxicity, nephrotoxicity, placental permeation

## Abstract

Drugs can be toxic to the fetus depending on the amount that permeates across the maternal–fetal barrier. One way to limit the amount which penetrates this barrier is to increase the molecular size of the drug. In this study, we have achieved this by encapsulating our model antibiotic (vancomycin hydrochloride, a known nephrotoxic agent) in liposomes. PEGylated and non-PEGylated liposomes encapsulating vancomycin hydrochloride were prepared using two different methods: thin-film hydration followed by the freeze–thaw method and the reverse-phase evaporation method. These liposomes were characterized by their hydrodynamic size and zeta potential measurements, CryoTEM microscopy, loading and encapsulation efficiency studies, in vitro release measurements and in vitro cytotoxicity assays using NRK-52 E rat kidney cells. We also determined the in vitro permeability of these liposomes across the human placental cell and dog kidney cell barriers. Vancomycin hydrochloride-loaded PEGylated liposomes (VHCL-lipo) of a size less than 200 nm were prepared. The VHCL-lipo were found to have the faster release of vancomycin hydrochloride and resulted in greater viability of NRK-52E cells. In vitro, the VHCL-lipo permeated the human placental cell and dog kidney cell barriers to a lesser extent than the free vancomycin hydrochloride. The data suggest a reduction in nephrotoxicity and permeability of vancomycin hydrochloride after encapsulation in PEGylated liposomes.

## 1. Introduction

A number of prescription and non-prescription drugs are used by pregnant women, leading to the risk of fetuses being exposed to drugs due to the transfer of drugs from the mother across the placental barrier. According to the Center for Disease Control and Prevention (CDC), most women (about 90%) take at least one medication during pregnancy, and 70% take at least one prescription medication [1]. With time, the average age of pregnancy is becoming close to 30, leading to an increase in consumption of a number of drugs consumed in pregnancy [2]. Often, the safety and selectivity of drug therapy during pregnancy are not the topmost priorities as far as drug design and development are concerned [3]. Owing to this, pregnant women are specifically excluded from clinical trial studies by pharmaceutical companies. The FDA previously classified the safety of medications in pregnancy as “A, B, C, D or X” (X = teratogenic/avoid) according to their teratogenicity potentials; however, these classifications have since been removed [4]. Hence, clinicians are forced to rely on primary medical literature for the safety of drugs during pregnancy.

Medication therapy during pregnancy exposes two individuals to the drugs because most of the pharmacological agents pass through the placenta [5]. Pharmacological agents can be toxic to the fetus depending on the amount it receives and owing to greater blood–brain barrier permeability and poor liver enzyme conjugating function [6]. The toxic effects caused by drugs on the fetus may lead to teratogenicity or congenital malformations. Therefore, alternative approaches to circumventing the fetal toxicity of the drug without compromising the efficacy to the mother are needed.

The mechanisms by which the drug can transfer across the placenta are passive diffusion, active transport, receptor-mediated uptake, endocytosis/pinocytosis, paracellular entry and placental metabolism [7]. Molecular size is an important physicochemical drug property that has a major influence on transplacental transfer. As the size of the drug increases, the chance that it passes through the placental barrier decreases, for example, blood cells or colloids.

One approach to restricting the transplacental transport of drugs is by increasing the molecular size of the drug. This can be achieved by either attaching the drug to some macromolecular carrier, e.g., cyclodextrins, or by using a colloidal drug (nano) delivery system such as liposomes for the delivery of the drug. Here, we propose the use of liposomes to tackle the problem of passage of vancomycin hydrochloride (VHCL) through the placental barrier. The goal is to create a drug formulation that can treat the mother while avoiding the fetus. Liposomes are spherical-shaped vesicles made up of phospholipids. The phospholipids possess a hydrophilic head group and a lipophilic tail that spontaneously self-assemble into bilayers, forming a sphere. Liposomes have been successfully employed in the past to encapsulate commonly utilized drugs, e.g., valproic acid [8], inulin [6], riboflavin, methotrexate, penicillin and indomethacin [9], and have been studied for their placental permeation. When the liposomal formulation of a drug is given intravenously, transplacental crossing is minimized. Warfarin, a teratogenic anticoagulant, decreased transplacental transfer by 57–66% after encapsulation in cationic multilamellar liposomes [10]. Valproic acid, an antiepileptic drug, was reduced by 32% in fetal circulation and 57% in placental tissue [8].

VHCL is a commonly used antibiotic during pregnancy for the treatment of severe sepsis. The nephrotoxicity of VHCL is very well-documented in the literature [11,12,13]. Because of the common use and importance of VHCL for life-threatening infections to the mother, it serves as a model drug for our investigation here. Although VHCL was previously FDA-classified as pregnancy category B, emerging data from our previous studies [14] suggest kidney damage in adults. Previously, transplacental crossing of VHCL was observed in women with amnionitis when administered over several days [15,16] and in-term pregnant women [17]. In rats and rabbits, VHCL was found to be toxic to maternal kidneys at higher doses [18]. Furthermore, data suggest that VHCL crosses the placental barrier [14], although early toxicity studies have not employed the more sensitive assessments of kidney function necessary to classify damage; thus, less is known about VHCL’s toxicity to the fetus. Our team has quantified the relationship between fetal VHCL exposure and kidney injury in rat pups at a dose of 250 mg/kg in a small sample (*n* = 6) [14]. VHCL was administered on Gestational Days (GD) 5, 6 and 7 (group I; Trimester 1); on GDs 12, 13 and 14 (group II; Trimester 2); and on GDs 18, 19 and 20 (group III; Trimester 3) [14]. The dams carried to term and delivered vaginally on GD 21. Kidneys were harvested from both dam and pup rats. The kidneys were homogenized, and following protein removal, samples were injected in LCMS/MS. As trimesters progressed, the deposition of VHCL increased linearly in rat dams as well as pups. The kidney tissue homogenate from rat dams was also subjected to the measurement of an extremely sensitive kidney injury marker: Kim-1. The results revealed an inverse relationship between trimesters and kidney damage as quantified by pup Kim-1. Trimester 1 had the highest level of Kim-1 compared to trimesters 2 and 3.

VHCL has previously been successfully encapsulated in liposomes. However, previous applications have been focused either on increasing the antibacterial efficacy of VHCL against resistant bacteria [19,20,21] or on the reduction in the kidney deposition of VHCL in animal models [22]. To make the VHCL more efficacious towards methicillin-resistant *Staphylococcus aureus,* additional helper lipids or polymers were added to the formulation. In this study, liposomes were designed to lessen the kidney accumulation of VHCL and hence to minimize its toxicity. We made liposomes with higher VHCL encapsulation efficacy. Our study differs from those previously reported because the aim of our study is to minimize the placental penetration to the fetus while being safe to the kidneys. To do this, we have formulated multivesicular vesicles (MVVs) liposomes with larger aqueous compartments. We hypothesize that placental and tubular transit can both be circumvented by using a colloidal drug delivery system such as a liposome for the delivery of the VHCL.

## 2. Materials

VHCL was purchased from Enzo (via VWR Enzo Life Science, San Jose, CA, USA) and demonstrated a purity of 99.3%. It is soluble in water >100 mg/mL, moderately soluble in methanol and insoluble in acetone, ether and chloroform. Acetonitrile, chloroform and methanol were purchased from VWR International (Radnor, PA, USA). Formic acid and PBS pH 7.4 were obtained from Fisher Scientific (Waltham, MA, USA). All solvents used were of HPLC or liquid chromatography tandem-mass spectrometry (LC/MS/MS) grade and obtained from Fisher Scientific (Waltham, MA, USA). Clinical-grade VHCL was utilized for animal studies and catheter retention studies (Hospira, Lake Forest, IL, USA). All phospholipids were purchased from NOF cooperation (White Plains, NY, USA). Cholesterol was purchased from Sigma Aldrich (Milwaukee, WI, USA). Clinical-grade VHCL was obtained as a gift to Dr Scheetz from Hospira.

## 3. Methods

### 3.1. Preparation of VHCL Loaded Liposomes

PEGylated and Non-PEGylated liposomes were prepared using (1) thin-film hydration followed by freeze–thaw and (2) reverse-phase evaporation methods, as described below. Both PEGylated and Non-PEGylated liposomes were similar in their composition, except for the use of 1,2-distearoyl-sn-glycero-3-phosphoethanolamine-N-[maleimide (polyethylene glycol)-2000] (PEG-DSPE 2000) for PEGylated liposomes. The molar ratio of DSPC:PEG-DSPE2000:Cholesterol was 1.85:0.15:1 for PEGylated liposomes and DSPC:Cholesterol was 2:1 for non-PEGylated liposomes.

Thin-Film Hydration followed by Freeze–Thaw Method: Liposomes were prepared using the thin-film hydration technique. Briefly, phospholipids were weighed out and dissolved in a mixture of methanol and chloroform (1:1, 9 mL each). The solution is then subjected to evaporation of organic phase using a Rotavac (Buchi, Essen, Germany) water bath kept at 55 °C. After complete evaporation of the solvents, the lipid film was further dried using nitrogen gas (~2 h). The resultant film was hydrated with phosphate buffer saline (PBS) pH 7.4 solution containing VHCL (12 mg/6 mL) to form the crude liposomes. Liposomes were subjected to 5 cycles of freeze–thaw in liquid nitrogen and were thawed in ambient conditions. The liposomes were gradually sized at around 200 nm using an extruder (Lipex Northern Lipids, Burnaby, BC, Canada) starting at 800 nm then 600 nm, 400 nm and 200 nm size membranes, once in each.

Reverse-Phase Evaporation Method: Phospholipids were weighed out and dissolved completely in a mixture of methanol and chloroform (1:1, 9 mL each). This solution was then mixed with VHCL (12 mg/6 mL) solution in PBS pH 7.4. This solution was sonicated for 5 min and then subjected to removal of the organic phase using a Rotavac (Buchi, Germany) water bath kept at 55 °C. It was subsequently exposed to nitrogen gas to facilitate further removal of the organic phase. Sizing using extrusion was performed using an extruder as described above.

The VHCL-loaded PEGylated liposomes prepared using thin-film hydration followed by freeze–thaw technique are abbreviated to VHCL-lipo here onwards.

### 3.2. Characterization of Liposomes

#### Dynamic Light Scattering (DLS) Measurements

The average hydrodynamic diameter and polydispersity index (PDI) of the liposome dispersions were determined by dynamic light scattering using a Malvern Zetasizer Nano (Malvern Instruments Ltd., Worcestershire, UK) at 25 °C using an argon-ion laser (488 nm) operating at 10.4 mW using non-invasive backscatter optics (NIBS). A total of 20 mL of the sample was diluted into 3 mL of PBS pH 7.4 in a cuvette to make the liposomes sufficiently dilute for analysis. For data analysis, the viscosity and refractive index of water were used. The system was calibrated with a polystyrene dispersion containing particles of 100 nm. The PDI is a measure of variation in particle size within a liposome population and varies from 0 (complete monodispersity) to 1 (large variations in particle size) and was calculated according to the method of Zhao et al. [23].

### 3.3. Zeta Potential Measurements

Electrophoretic mobility measurements (Zetasizer Nano-Z, Malvern Instruments, Malvern, UK) were performed after dilution of the liposomes in PBS pH 7.4. The instruments were calibrated using polystyrene latex beads of defined zeta potential. The same sample dilution used for particle size analysis was then transferred from the cuvette to a disposable folded capillary cell (DTS1070) (Malvern, UK) for zeta potential measurement.

#### 3.3.1. Examination of Vesicle Morphology with Cryo-TEM

JEOL 1230 TEM with an ACD and cryo holder (Jeol, Akishima, Japan) was used to study the morphology of the formed liposomal vesicles. Briefly, liposomes were loaded onto Lacey Formvar/Carbon, 300 mesh, copper grids, and the thin aqueous films were blotted with filter paper and immediately plunged into liquid ethane using Vitrobot FEI (Hillsboro, OR, USA). The frozen grids were stored in liquid nitrogen and transferred to a cryotransfer holder (Gatan (Pleasanton, CA, USA)) under liquid nitrogen at approximately −180 °C. Images were recorded on a Gatan Orius bottom-mount CCD camera (Pleasanton, CA, USA) using a 100 kV accelerating voltage.

#### 3.3.2. Determination of Loading Efficiency of VHCL into Liposomes by HPLC

The amount of VHCL loaded into the liposomal suspension was determined by HPLC. A standard curve using the known concentrations of VHCL was plotted. A stock solution of VHCL with a 1.5 mg/mL concentration was prepared by dissolving 15 mg of VHCL in a mixture of 5 mL DI water and 5 mL methanol. Subsequently, concentrations of 2, 5, 10, 20, 30, 40 and 50 μg/mL VHCL in water for HPLC were prepared from a stock solution. The VHCL content was analyzed by Agilent 1200 High-Pressure Liquid Chromatography (HPLC) with ChemStation software (version Rev. B. 04.03). The method utilized a Kinetex Biphenyl column 2.6 mm, a 50 × 3 mm column (Phenomenex, Torrance, CA, USA) and a gradient of acetonitrile (VWR International, Radnor, PA, USA) from 0% to 30% within 5 min and VHCL was eluted with 0.1% formic acid [24]. VHCL was detected at 198 nm. The loading efficiency was calculated by comparing the concentration of VHCL added during the formation of the liposomes (12 mg/6 mL) with the actual concentration of VHCL obtained in the liposome suspension after extrusion using HPLC, using the following equation:

$$Percentage\;Loading=\left(\frac{Actual\;concentration\;in\frac{mg}{mL}as\;determined\;by\;HPLC\;}{Theoretical\;Concentration\;in\;mg/mL}\right)\times 100\%$$

#### 3.3.3. Determination of Encapsulation Efficiency of VHCL into Liposomes by HPLC

The amount of VHCL encapsulated within liposomes was determined by ultracentrifugation (Beckman Coulter, Brea, CA, USA) of 500 µL of the liposomal solution for 30 min at 186,000 g at 4 °C. The supernatant was separated from the pellet, and the pellet was subsequently resuspended in PBS pH 7.4. To lyse the liposomes and release VHCL, the pellet was dissolved in equal parts of methanol and thoroughly mixed. The VHCL content in the supernatant and pellet was analyzed by HPLC, as described above. Encapsulation efficiency was calculated by comparing the concentration of vancomycin hydrochloride in the pellet to the loading efficiency:

$$Percentage\;Encapsulation=\left(\frac{Concentration\;in\frac{mg}{mL}of\;VHCL\;in\;the\;pellet}{Loading\;concentration\;in\frac{mg}{mL}of\;VHCL}\right)\times 100\%$$

#### 3.3.4. In Vitro Release of VHCL from VHCL-Loaded Liposomes

VHCL release from liposomes was studied using the dialysis bag method. Six milliliters of liposomes containing 12 mg of VHCL was dialyzed (50 kD molecular weight cut off, Spectrum Laboratories, Rancho Dominguez, CA, USA) against 50 mL PBS pH 7.4. Aliquots of 500 μL of the buffer were taken for HPLC analysis at time intervals of 0 h, 5 min, 15 min, 30 min, 1 h, 2 h, 3 h, 6 h, 12 h, 24 h and 48 h. The removed amount of buffer was replaced with 500 μL of PBS pH 7.4. Aliquots were analyzed for VHCL content by HPLC as described in the earlier section. No separation of the encapsulated drug from the unencapsulated drug was performed during this experiment or all experiments described henceforth.

#### 3.3.5. In Vitro Cytotoxicity Assay

To evaluate cytotoxicity, NRK-52 E cells (rat epithelial kidney cells, ATCC CRL-1571) were treated over a period of 8, 24, 48 and 72 h on a 96-well plate after plating the cells at a density of 1000 cells/well. The VHCL concentrations used varied and were 2.7 mg/mL, 3.4 mg/mL and 4.3 mg/mL. Following the treatment, viability was measured using Alamar Blue assay. The treatment groups included VHCL and VHCL-lipo, blank liposomes and cells with no treatment.

#### 3.3.6. Permeation Assay

BeWo cells (human placental cells, CCL-98 ATCC) and MDCK cells (cells were isolated from normal kidney tissue from a normal, adult, female cocker spaniel, CCL-34, ATCC) were plated on collagen-coated inserts of Transwell plates at a concentration of 10^6^ cells/well. Trans Epithelial Electrical Resistance (TEER) measurements were taken until a stable plateau was reached (usually after 7 days). A Lucifer Yellow assay was run in one of the Transwell plates to confirm the quality, tightness and integrity of the barrier. To assess the permeability; the in vitro barrier was treated with VHCL (1 mg/mL Enzo and Hospira) and VHCL-lipo (1 mg/mL) over a period of 12, 24 or 48 h on a 24-well plate (*n* = 3). Following the treatment, the permeability of the treatment groups was assessed by running the media samples from the basal side of the Transwell taken at various time points using HPLC. VHCL-lipos used in this experiment were used as prepared and the encapsulated drug was not separated from the unencapsulated. Percentage permeation was calculated using the following formula:

$$\%\;Permeation=\frac{VHCL\;concentration\;in\;mg/mL\;in\;basal\;sample\;}{1\;mg/mL}\times 100\%$$

#### 3.3.7. Statistical Analysis

The results were expressed as the mean ± standard deviation. Most experiments were repeated at least three times. Statistical significance of differences between groups was calculated using the Student’s *t*-test or two-way ANOVA using GraphPad Prism, Version 9.3.1. *p* values < 0.05 were considered statistically significant.

## 4. Results

### 4.1. Characterization of Liposomes

**Figure 1:**
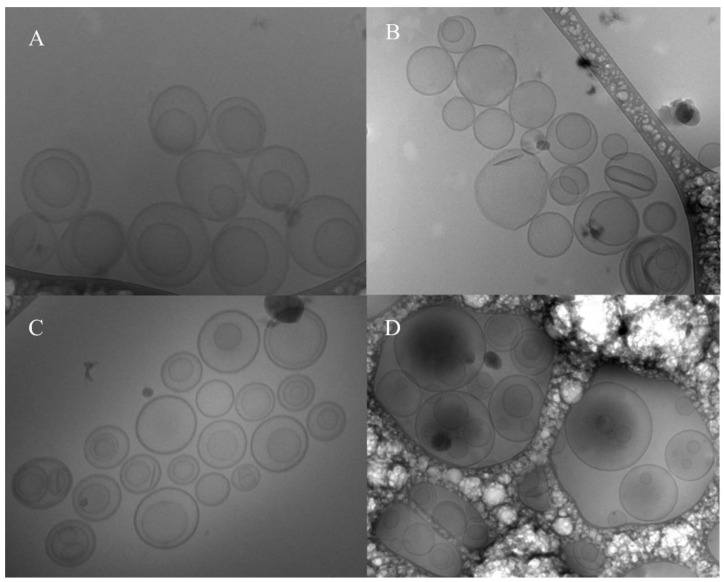
Cryo TEM images of liposomes: (**A**) PEGylated Reverse-Phase Evaporation, (**B**) Non-PEGylated Reverse-Phase Evaporation, (**C**) Non-PEGylated Freeze–Thaw, (**D**) PEGylated Freeze–Thaw.

Liposomes made using both thin-film hydration followed by freeze–thaw and the reverse-phase evaporation method had a PDI of less than 0.2, and the mean diameter for all the liposomes was less than 200 nm (Table 1). This demonstrates the formation of a homogenized vesicle size for both the PEGylated and Non-PEGylated liposomes. The Cryo-TEM images confirmed the results obtained using the Zetasizer and depicted the differences in morphology because of the varying processing conditions in preparing the liposomes, viz., thin-film hydration followed by freeze–thaw and reverse-phase evaporation techniques (Figure 1). The liposomes produced using thin-film hydration followed by freeze–thaw (Figure 1A,C) had concentric phospholipid bilayers and had liposomes within a liposome, i.e., a multivesicular vesicle (MVV) structure. Thus, the Cryo-TEM images also depict a distinct morphological difference between liposomes produced using these two methods, as seen in Table 1. PEGylated freeze–thaw liposomes showed loading and encapsulation percentages of 40% ± 2.98 and 62% ± 0.8, respectively. Compared to them, all other types of liposomes, for example, non-PEGylated liposomes, prepared using freeze–thaw or reverse-phase evaporation, had lower loading and encapsulation.

### 4.2. In Vitro Release of VHCL from Liposomes

**Figure 2:**
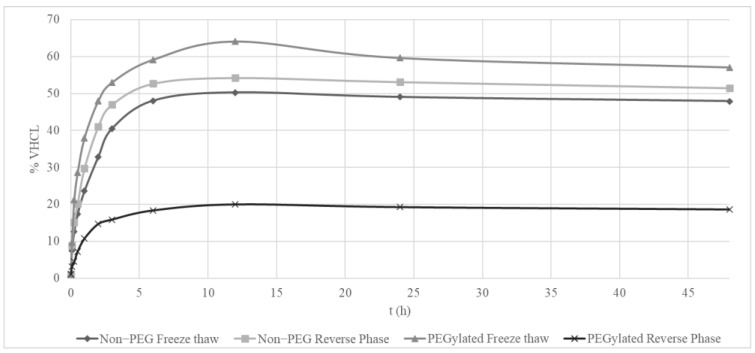
Concentration of vancomycin hydrochloride (VHCL) released over a period of time (min) in vitro in a dialysis bag experiment (*n* = 1).

The data analyzed from the 48 h release studies highlighted that PEGylated reverse-phase liposomes were fast compared to the rest at releasing the load of VHCL. PEGylated freeze–thaw liposomes were the slowest in releasing the load compared to other types of liposomes (Figure 2). PEGylated liposomes prepared using thin-film hydration followed by the freeze–thaw method also provided the highest release rate of about 64%. The slowest release rate is shown to be from the PEGylated reverse-phase liposomes with the max concentration release of 20%. The difference in the release rate was about 3.25-fold.

### 4.3. Cytotoxicity Assay

**Figure 3:**
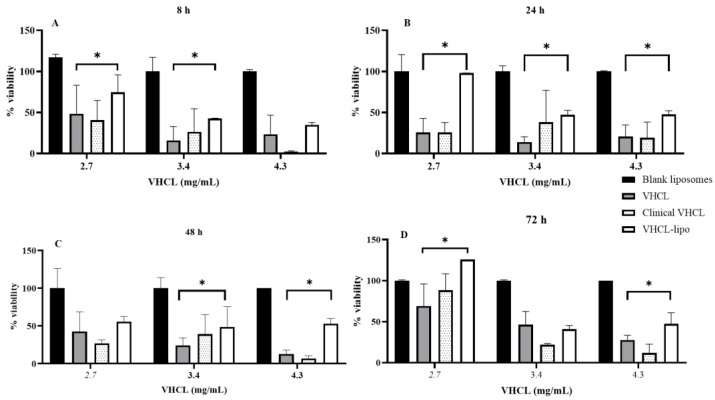
Percentage viability of NRK-52 E cells (*n* = 6) on treatment with various concentrations of VHCL-loaded PEGylated liposomes (VHCL-lipo) vs. vancomycin hydrochloride (VHCL) unencapsulated at various time points: 8 h (**A**), 24 h (**B**), 48 h (**C**) and 72 h (**D**). Data represented as mean ± SD (* *p* < 0.0001 VHCL-lipo vs. VHCL two-way ANOVA). Data normalized for non-treated cells for each time point.

A concentration- and time-dependent reduction in viability was observed after treatment of NRK-52 cells with either unencapsulated VHCL or VHCL-lipo. All dosing concentrations of VHCL-loaded liposomes resulted in greater viability of the cells at 48 h (*p* < 0.0001 for VHCL lipo vs. VHCL, *p* = 0.002 for VHCL lipo vs. Clinical VHCL and *p* = 0.029 for VHCL lipo vs. blank) and 72 h (*p* = 0.0011 for VHCL lipo vs. VHCL, *p* = 0.0003 for VHCL lipo vs. Clinical VHCL and *p* < 0.0001 for VHCL lipo vs. blank), as depicted in Figure 3.

### 4.4. Permeation Assay

**Figure 4:**
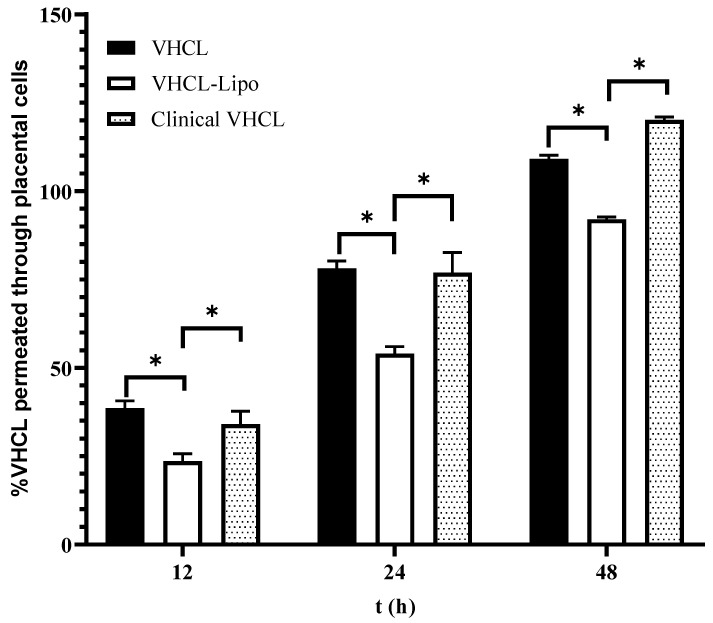
In vitro percentage cumulative placental (BeWo cells) permeation vs. time of exposure (*n* = 6) of cells to VHCL-loaded PEGylated liposomes prepared using thin-film hydration followed by the freeze–thaw method (VHCL-lipo) vs. vancomycin hydrochloride (VHCL) vs. Clinical VHCL formulation at a concentration of 1 mg/mL. Data represented as mean ± SD; (* *p* < 0.0001 VHCL-lipo vs. VHCL and VHCL-lipo vs. Clinical VHCL) Two-way ANOVA.

In the permeation studies using the placental cells (BeWo), there was a statistically significant difference between the permeation of VHCL-lipo and VHCL for all time points assessed (*p* < 0.0001). VHCL-lipo had a lower percentage permeation than the Clinical VHCL and VHCL for 12 h, 24 h and 48 h (Figure 4). Between 24 and 48 h, there was almost a 1.5-fold difference between VHCL and Clinical VHCL.

**Figure 5:**
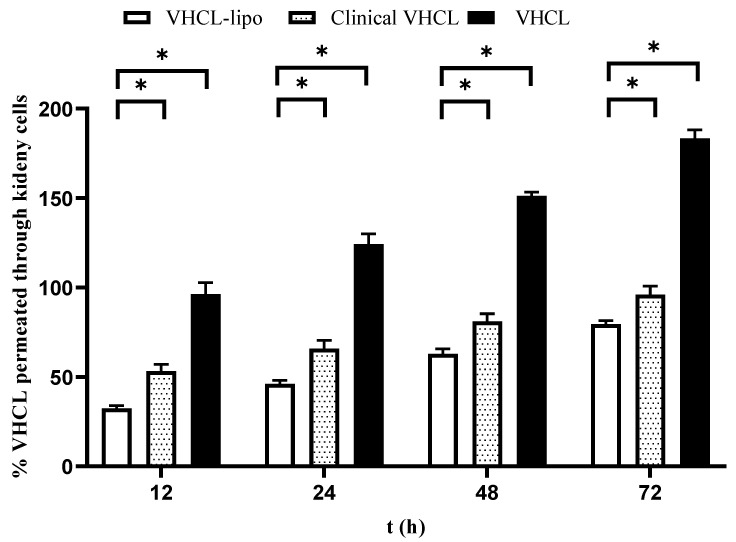
In vitro percentage cumulative permeation through kidney cells (MDCK cells) vs. time of exposure of cells to vancomycin hydrochloride (VHCL)-loaded PEGylated liposomes prepared using thin-film hydration followed by the freeze–thaw method (VHCL-lipo) vs. vancomycin hydrochloride (VHCL) vs. Clinical VHCL formulation at a concentration of 1 mg/mL (*n* = 6). Data represented as mean ± SD; (* *p* < 0.0001 VHCL-lipo vs. Clinical-VHCL) Two-way ANOVA.

For permeation studies in the kidney cells (MDCK), there was a statistical percentage permeation difference (almost twofold) between the VHCL-lipo and Clinical VHCL for all time points evaluated (*p* < 0.0001) (Figure 5). Interestingly, in the kidney permeation experiment, VHCL permeated the least compared to Clinical VHCL and VHCL-lipo.

## 5. Discussion

A nanosized liposomal preparation of VHCL was obtained using two methods of encapsulation, viz., thin-film hydration followed by freeze–thaw and reverse-phase encapsulation. Various methods of encapsulation have been suggested in the literature for hydrophilic compounds [25] such as VHCL. PEGylated liposomes prepared using thin-film hydration followed by the freeze–thaw method provided the highest encapsulation as well as loading when compared to the rest. The thin-film hydration followed by the freeze–thaw method may be superior for VHCL encapsulation because of the formation of multivesicular vesicles (liposomes within liposomes), resulting in an increased volume for the internal aqueous compartment wherein the water-soluble encapsulated drug is present. As seen from the TEM images of liposomes produced using the thin-film hydration followed by freeze–thaw method (Figure 1C,D), the repeated cycles of freezing and thawing of MVVs produced a physical disruption of the liposomal phospholipid bilayers because of the formation of ice crystals during the freezing process, which broke apart the closely spaced lamellae of the vesicles. This could lead to an increase in the ratio of aqueous solute to lipid, resulting in a higher loading efficiency. Cullis et al. found that when MVV preparations were subjected to five cycles of freeze on liquid nitrogen followed by thawing in warm water, liposomes of high encapsulation efficiency (up to 88%) could be obtained. Freeze-fracture electron micrographs revealed vesicles within vesicles [26]. The reverse-phase evaporation, on the other hand, produces a high aqueous space to lipid ratio, allowing the creation of large amounts of hydrophilic drugs [27], but could not yield as high encapsulation as with the freeze–thaw method. In the in vitro release study, the lowest release was obtained from PEGylated reverse-phase VHCL-lipo. This could be due to the lowest percentage loading in this type of liposome (Table 1). The magnitude of VHCL released from distinct types of liposomes is related to the amount encapsulated as depicted in Table 1. Hence, here onwards, only PEGylated liposomes (VHCL-lipo) made with thin-film hydration followed by the freeze–thaw method were used to conduct all remaining experiments.

In the in vitro cytotoxicity experiments, the empty liposomes (without any VHCL encapsulated in them) showed 100% viability as expected considering the well-known nontoxicity of phospholipids. VHCL-lipo showed the highest dose-dependent viability (higher viability for 2.7 mg/mL than 4.3 mg/mL for 8, 24, 48 and 72 h) compared to the unencapsulated form and the clinically used VHCL formulation. This could be due to the sustained release of VHCL through the encapsulated multi-layers so that the cell is not exposed to a higher concentration of VHCL for a prolonged period which leads to higher viability.

In the permeation studies using placental cells (BeWo) and kidney cells (MDCK), VHCL-lipo permeated less over a period of time than the free VHCL. The reason for the lower permeation of the liposomal formulation of VHCL could be due to resistance of its passage through the in vitro barrier by virtue of the size and surface PEGylation. Higher overall percentage permeation with VHCL-lipo was observed in the case of the placental barrier compared to the kidney barrier, suggesting the kidney barrier might be more impermeable than the placental barrier to VHCL-lipo. This further confirms our hypothesis that the encapsulation of VHCL in liposomes leads to a reduced placental transfer as well as to reduced nephrotoxicity. Interestingly, the currently marketed clinical formulation of VHCL also permeated less compared to the unencapsulated VHCL, most likely due to the excipient used, e.g., PEG [28].

## 6. Conclusions

PEGylated liposomes encapsulating VHCL were successfully prepared. The results highlight a reduction in VHCL permeation via the placental barrier and kidney barrier after encapsulation in PEGylated liposomes with minimal toxicity to the kidney cells in vitro.

## Figures and Tables

**Table 1:** Percentage loading and percentage encapsulation for the four types of liposomes (*n* = 3).

Type of Liposomes	Percentage Loading	Percentage Encapsulation
Non-PEGylated liposomes prepared by thin-film hydration followed by freeze–thaw	29 ± 2.44	15.5 ± 0.9
Non-PEGylated liposomes prepared by reverse-phase evaporation	32 ± 1.76	28.5 ± 1
PEGylated liposomes prepared by thin-film hydration followed by freeze–thaw	40 ± 2.98	62 ± 0.8
PEGylated liposomes prepared by reverse-phase evaporation	24 ± 2.86	16.9 ± 0.92

## Abbreviations

VHCL	vancomycin hydrochloride
VHCL-lipo	PEGylated vancomycin hydrochloride
MVV	Multivesicular vesicles
MDCK	Madin–Darby Canine Kidney cells
BeWo	BeWo is a cell line exhibiting epithelial morphology that was isolated from the placenta of a patient with choriocarcinoma
NRK-52E	Rat Kidney epithelial cells
TEER	Trans Epithelial Electrical Resistance
CDC	Center for Disease Control and Prevention
LCMS/MS	Triple-quad Liquid chromatography-mass spectrometry
Kim-1	Kidney Injury molecule-1
GD	Gestational Day
FDA	Food and Drug Administration
PBS	Phosphate buffer saline pH 7.4
ATCC	American-Type Culture Collection
TEM	Transmission electron Micrography
PDI	Polydispersity Index
PEG	Polyethylene glycol
PEG-DSPE 2000	(1,2-distearoyl-sn-glycero-3-phosphoethanolamine-N- [maleimide (polyethylene glycol)-2000])
HPLC	High-pressure liquid chromatography
CCD	Charge-Coupled Device
ACD	Air Conditioning Disconnect
